# Exploring multivariate clinical chemical routine data concerning three major disease groups

**DOI:** 10.1155/S146392468800015X

**Published:** 1988

**Authors:** Jan B. Hemel, Hilko van der Voet, Rolf Hendriks, Frans R. Hindriks, Willem van der Slik

**Affiliations:** 1 Central Laboratory for Clinical Chemistry University Hospital Groningen P.O. Box 30001 Groningen NL-9700 RB The Netherlands; 2 Research Group Chemometrics Pharmaceutical Laboratories State University of Groningen A. Deusinglaan 2 Groningen NL-9713 AW The Netherlands; 3 Agricultural Mathematics Group P.O. Box 100 Wageningen NL-6700 AC The Netherlands

## Abstract

In preparation for multivariate analysis, an exploratory study has been undertaken to investigate the relative position, separability, homogeneity and shape of three major disease groups, using data from a clinical chemical routine package.

The data set consists of 46 hepatology patients, 50 nephrology patients and 46 cardiology patients, and the measured blood levels include 20 common clinical chemical routine assays. Missing value problems were avoided by deleting some of the variables and objects.

A univariate analysis was used as the basis ofa rescaling of the data.

Bivariate (pairwise) plots of some major assays each show limited separation. The set of three such plots of the three major principal components reveals more distinction between the groups than was offered by univariate analysis. Three-dimensional extensions of these techniques allow better insight than any of the two-dimensional plots, but these three-dimensional versions require more plots for complete interpretation.

Non-linear mapping of the data is the best way of retaining the distances and a fairly good separation is achieved in the plot. The plot is less informative about shape and relative position of the classes.

Representation of the data as pictures of faces does not offer additional information and visual clustering is worse than in any of the techniques mentioned.

During the analysis many assumed properties of the data are confirmed and a good starting pointfor multivariate classification is attained. Easy visual detection of outliers is offered by all techniques. Unfortunately, valuable information is lost in this data set by deleting some incomplete variables.


Journal of Automatic Chemistry, Vol. 10, No. 2 (April–June 1988), pp. 67–78

### Exploring multivariate clinical chemical routine data concerning three major disease groups

**Jan B. Hemel,**

*Central Laboratory for Clinical Chemistry, University Hospital Groningen, P.O. Box 30001, NL-9700 RB Groningen, The Netherlands*

**Hilko van der Voet*,**

*Research Group Chemometrics, Pharmaceutical Laboratories, State University of Groningen, A. Deusinglaan 2, NL-9713 AW Groningen, The Netherlands*

**Rolf Hendriks, Frans R. Hindriks and Willem van der Slik**

*Central Laboratory for Clinical Chemistry, University Hospital Groningen, P.O. Box 30001, NL-9700 RB Groningen, The Netherlands*

*In preparation for multivariate analysis, an exploratory study has been undertaken to investigate the relative position, separability, homogeneity and shape of three major disease groups, using data from a clinical chemical routine package.*

*The data set consists of 46 hepatology patients, 50 nephrology patients and 46 cardiology patients, and the measured blood levels include 20 common clinical chemical routine assays. Missing value problems were avoided by deleting some of the variables and objects.*

*A univariate analysis was used as the basis of a rescaling of the data.*

*Bivariate (pairwise) plots of some major assays each show limited separation. The set of three such plots of the three major principal components reveals more distinction between the groups than was offered by univariate analysis. Three-dimensional extensions of these techniques allow better insight than any of the two-dimensional plots, but these three-dimensional versions require more plots for complete interpretation.*

*Non-linear mapping of the data is the best way of retaining the distances and a fairly good separation is achieved in the plot. The plot is less informative about shape and relative position of the classes.*

*Representation of the data as pictures of faces does not offer additional information and visual clustering is worse than in any of the techniques mentioned.*

*During the analysis many assumed properties of the data are confirmed and a good starting point for multivariate classification is attained. Easy visual detection of outliers is offered by all techniques. Unfortunately, valuable information is lost in this data set by deleting some incomplete variables.*

### Introduction

During the past decades the number of constituents that can be measured in body fluids has increased substantially. Simultaneously, the costs per assay decreased at an even more rapid rate due to large-scale laboratory automation. These facts have stimulated the physician to order more assays than can be effectively interpreted by a human without help from sophisticated techniques. Although each assay may contribute some information, the sequential univariate way of interpreting the results leaves part of the information unrevealed. Fortunately, the *impasse* that results can be broken.

Firstly, the physician could be advised to order only those assays that will give him the desired information, in other words, to order very *selectively*. This requires a thorough knowledge of the value of the assays for each diagnosis. As clinical chemistry offers ever more new and possibly valuable assays, it is difficult to keep this knowledge up to date. The introduction of protocols of diagnosis and treatment in medicine, supported by recent medical decision-making techniques, is an attempt to optimize the use of laboratory results by selection of an optimal subset of all assays for a specific problem. Advantages of this approach include reduction of overall costs and growing experience with a selection of assays, leading to assessment of their value.

Another approach is to change the way of interpreting the results from sequentially univariate (the assay outcomes are judged successively) to multivariate (all assays are judged simultaneously). As the maximum number of features that one can simultaneously grasp does not exceed three by much, the wealth of data coming from a modern screening series of, say 20, laboratory assays is far too large to be interpreted without help. This is where statistical multivariate techniques may be useful. By representing a patient's record as a point in a $p$-dimensional space spanned by the $p$ assays as axes, the results of all $p$ assays can be judged at once. Mathematical models enveloping each disease class may be developed, and this can be followed by classification of an unknown test point into the class whose model fits best. Furthermore, multivariate processing of data may indicate which assays contain most discriminatory information between disease classes and thus supplies suggestions about which measurements should be done to clear what doubts.

In this way, multivariate data analysis may both reduce the number of assays needed by maximizing the uncovered information, and indicate which assays offer most.

Since medical diagnosis is in fact a kind of classification, multivariate classification is the most interesting branch of multivariate analysis of medical data. It is stressed that classification is not necessarily choosing the one and only diagnosis, but may very well take the form of a

* Present address: Agricultural Mathematics Group, P.O. Box 100, NL-6700 AC Wageningen, The Netherlands.

probabilistic differential diagnosis. Multivariate classification is an often-occurring aim of data analysis [see, for instance, 1–8]. Although at first sight the procedure may seem to consist only of choosing a multivariate classification method (MCM) and applying it to the data using an appropriate computer program, in most cases many unexpected problems are encountered. Problems of scaling, transformation, outlier detection, class modelling, and many more will trouble the investigator and may prevent him from reaching his goal. Some of these problems may (and should) be foreseen and even solved by exploring the data before starting the actual classification procedure. A carefully executed exploration will answer questions about separability, relative position and shape of the classes, it may suggest adequate class models, and it will indicate possible outliers. Tukey has written a comprehensive work about data exploration [9].

Before starting with the exploration it is useful to preprocess the data by eliminating missing values and scaling the variables [2, 7, 8 and 10].

The actual exploration includes several stages.

In the first stage univariate statistics, like means and moments, may be calculated for the complete data set as well as for each class separately. These will detect clear separability on a single variable. Histograms and other graphical displays may add univariate distributional information. The knowledge of the univariate separability that can be acquired in this way gives an impression of the multivariate distinction as well. However, bad univariate separation does not imply multivariate overlap, making a multivariate approach useful.

The second step involves calculation of correlations. These provide us with an impression of the dispensability of each variable in the presence of another one.

In the third stage, several display techniques that aim at retaining the multivariate aspect of information in the data complete the exploration.

This paper is dedicated to the exploration of a medical dataset, consisting of clinical chemical screening data of patients suffering from a heart, liver or kidney disease. Usually, in medical diagnosis a sequential univariate approach is used implicitly in the application of reference intervals [11]. Multivariate diagnosis is expected to use a larger part of the information present in the data. To investigate the separability, relative positions and shape of these major disease groups, and to detect the presence of atypical cases, this study was undertaken.

After the description of the computer equipment and the programs that were used the data set is introduced. In the following section a selection of multivariate display methods is discussed. After a section discussing the necessary preprocessing of the data the main section will contain a discussion of exploration results of the data. The paper is concluded with an overview of the information that is derived from the various techniques.

### Equipment and computer programs

All samples were analysed on a Technicon SMAC continuous flow analyser. Calculations were done on Groningen University's Control Data Cyber 170/760 computer. Plots were made on the University's Versatec V80 electrostatic plotter.

The following programs were used:

(1) SIMCA-3B [12], for the calculation of modelling powers.

(2) ARTHUR [13], for variable selection, non-linear mapping and principal-components plots.

(3) Some *ad hoc* programs for the three-dimensional plots.

(4) CLAS [14], for all other calculations.

### Data

Most multivariate studies aim at distinguishing between very similar groups, using a highly selected set of very specific assays. However, classification into major disease groups using only clinical chemical routine data can serve as the first step in referring a patient to the right medical specialist. Although separation of major disease groups may at first seem trivial, the heterogeneity within these large groups and cases with multiple diseases make separation difficult.

The data set that is explored consists of 142 patient records. The patients suffered from the following disease groups: 46 liver diseases (15 alcoholic cirrhosis, five primary biliary cirrhosis, 26 cirrhosis due to chronic active hepatitis – the LIVER class), 50 kidney diseases (various, unspecified – the KIDNEY class), 46 heart diseases (24 myocardial infarction, 22 coronary artery disease – the HEART class).

The criteria for selection of the patients were: (1) diagnosis certain within the boundaries of the class; (2) no concurrent disease from any of the two other classes present; (3) admitted to hospital in 1982; (4) blood sample available within 10 days of admittance.

The only information about the patients, apart from their diagnostic class, consists of their blood concentrations of sodium, potassium, chloride, urea, creatinine, uric acid, alkaline phosphatase (AP), lactate dehydrogenase (LDH), aspartate aminotransferase (ASAT), alanine aminotransferase (ALAT), total bilirubin, direct bilirubin, calcium, inorganic phosphate, total protein, albumen, cholesterol, triglycerides, iron and $\gamma$-glutamyl transferase (GGT), expressed in appropriate units. This set of assays is the routine clinical chemical package used at Groningen University Hospital. It has not been optimized for discrimination between HEART, LIVER or KIDNEY patients. For each patient the complete series of assays was performed on a single blood sample, taken within 10 days of admission. Twelve per cent of the values in the data is missing due to a selective ordering of assays by the physican. More details about the data are given in table 1.

*Table 1. Summary of the data set before and after reduction because of missing values.*

| | | | With missing values | | | Without missing values | |
|---|---|---|---|---|---|---|---|
| Variable | Unit | Class | Mean | s.d. | % miss. | Mean | s.d. |
| Sodium | mMol/l | heart | 139 | 2·6 | 4 | 139 | 2·6 |
| | | liver | 138 | 3·8 | 4 | 137 | 4·2 |
| | | kidney | 138 | 4·7 | 0 | 137 | 4·7 |
| | | all | 138 | 3·9 | 3 | 138 | 4·0 |
| Potassium | mMol/l | heart | 4·3 | 0·47 | 2 | 4·2 | 0·46 |
| | | liver | 4·0 | 0·50 | 7 | 3·9 | 0·53 |
| | | kidney | 4·6 | 0·62 | 2 | 4·5 | 0·64 |
| | | all | 4·3 | 0·59 | 4 | 4·3 | 0·60 |
| Chloride | mMol/l | heart | 103 | 2·6 | 4 | 103 | 2·7 |
| | | liver | 102 | 6·2 | 2 | 101 | 6·6 |
| | | kidney | 102 | 6·6 | 4 | 101 | 6·8 |
| | | all | 102 | 5·4 | 4 | 102 | 5·7 |
| Urea | mMol/l | heart | 6 | 1·9 | 0 | 6 | 2·0 |
| | | liver | 6 | 5·7 | 0 | 6 | 6·3 |
| | | kidney | 17 | 12·4 | 0 | 18 | 12·9 |
| | | all | 10 | 9·5 | 0 | 11 | 10·2 |
| Creatinine | μMol/l | heart | 93 | 14 | 0 | 94 | 15 |
| | | liver | 84 | 61 | 0 | 88 | 68 |
| | | kidney | 384 | 389 | 2 | 407 | 402 |
| | | all | 191 | 270 | 1 | 207 | 289 |
| *Uric acid* | *mMol/l* | *heart* | *0·36* | *0·08* | *41* | — | — |
| | | *liver* | *0·29* | *0·10* | *11* | — | — |
| | | *kidney* | *0·42* | *0·12* | *6* | — | — |
| | | *all* | *0·36* | *0·12* | *19* | — | — |
| AP | U/l | heart | 87 | 27 | 2 | 89 | 27 |
| | | liver | 238 | 219 | 9 | 251 | 232 |
| | | kidney | 101 | 54 | 2 | 104 | 57 |
| | | all | 139 | 143 | 4 | 144 | 151 |
| LDH | U/l | heart | 326 | 245 | 9 | 334 | 256 |
| | | liver | 280 | 140 | 0 | 292 | 153 |
| | | kidney | 260 | 86 | 4 | 263 | 88 |
| | | all | 287 | 168 | 4 | 295 | 178 |
| ASAT | U/l | heart | 48 | 64 | 0 | 54 | 69 |
| | | liver | 118 | 150 | 2 | 127 | 166 |
| | | kidney | 25 | 27 | 0 | 25 | 29 |
| | | all | 62 | 101 | 1 | 66 | 109 |
| ALAT | U/l | heart | 25 | 12 | 2 | 25 | 12 |
| | | liver | 105 | 98 | 4 | 99 | 87 |
| | | kidney | 27 | 54 | 0 | 28 | 58 |
| | | all | 51 | 74 | 2 | 49 | 68 |
| *Bilirubin, tot.* | *μMol/l* | *heart* | *9* | *6* | *52* | — | — |
| | | *liver* | *64* | *141* | *7* | — | — |
| | | *kidney* | *8* | *7* | *14* | — | — |
| | | *all* | *30* | *93* | *24* | — | — |
| *Bilirubin, dir.* | *μMol/l* | *heart* | *1·5* | *0·7* | *46* | — | — |
| | | *liver* | *32·5* | *80·6* | *2* | — | — |
| | | *kidney* | *1·9* | *1·4* | *14* | — | — |
| | | *all* | *14·0* | *52·8* | *20* | — | — |
| *Calcium* | *mMol/l* | *heart* | *2·4* | *0·14* | *26* | — | — |
| | | *liver* | *2·3* | *0·16* | *4* | — | — |
| | | *kidney* | *2·4* | *0·21* | *2* | — | — |
| | | *all* | *2·3* | *0·19* | *11* | — | — |
| *Phosphate* | *mMol/l* | *heart* | *1·0* | *0·26* | *39* | — | — |
| | | *liver* | *1·1* | *0·30* | *4* | — | — |
| | | *kidney* | *1·5* | *0·66* | *0* | — | — |
| | | *all* | *1·2* | *0·51* | *14* | — | — |
| *Protein* | *g/l* | *heart* | *66* | *5·4* | *11* | — | — |
| | | *liver* | *65* | *9·3* | *0* | — | — |
| | | *kidney* | *62* | *9·6* | *2* | — | — |
| | | *all* | *64* | *8·6* | *4* | — | — |

*Table 1 continued.*

| | | | With missing values | | | Without missing values | |
|---|---|---|---|---|---|---|---|
| Variable | Unit | Class | Mean | s.d. | % miss. | Mean | s.d. |
| *Albumen* | *g/l* | *heart* | *44* | *2·9* | *39* | — | — |
| | | *liver* | *35* | *5·7* | *4* | — | — |
| | | *kidney* | *36* | *7·4* | *2* | — | — |
| | | *all* | *38* | *7·0* | *15* | — | — |
| *Cholesterol* | *mMol/l* | *heart* | *7* | *1·6* | *24* | — | — |
| | | *liver* | *5* | *2·7* | *4* | — | — |
| | | *kidney* | *6* | *2·8* | *24* | — | — |
| | | *all* | *6* | *2·6* | *18* | — | — |
| *Triglycerides* | *mMol/l* | *heart* | *2·4* | *1·1* | *61* | — | — |
| | | *liver* | *1·5* | *1·0* | *15* | — | — |
| | | *kidney* | *2·2* | *1·4* | *36* | — | — |
| | | *all* | *1·9* | *1·2* | *37* | — | — |
| *Iron* | *μMol/l* | *heart* | *14* | *8·2* | *54* | — | — |
| | | *liver* | *19* | *10·1* | *11* | — | — |
| | | *kidney* | *14* | *8·0* | *20* | — | — |
| | | *all* | *16* | *9·2* | *28* | — | — |
| *GGT* | *U/l* | *heart* | *39* | *32* | *50* | — | — |
| | | *liver* | *372* | *632* | *15* | — | — |
| | | *kidney* | *51* | *123* | *16* | — | — |
| | | *all* | *169* | *423* | *27* | — | — |

Numbers of patients initially:
$N_{heart}=46$; $N_{liver}=46$; $N_{kidney}=50$; $N_{total}=142$;
After removing missing values:
$N_{heart}=38$; $N_{liver}=36$; $N_{kidney}=43$; $N_{tot}=117$
*Italics*: variable is deleted after missing value removal.

### Multivariate display methods

To get information about the separability of the classes, visual inspection of the multidimensional space and the situation of the object patterns in that space can be a helpful tool. Since human imagination can only cope with spaces with up to three dimensions, so-called display methods map the multidimensional data onto a low (usually two) dimensional space.

What could be the use of display methods? One may get an impression of the separability of the classes, of the presence of outliers or atypical cases, and, if mathematical models are fitted, one may visually judge the models. It is stressed that separability need not – and perhaps should not – be the only purpose of exploring data.

To reach these goals there are some requirements to be fulfilled. (1) distances between objects (and classes) should be conserved as well as possible; (2) the information content, in the sense of variance retained in the picture, should be as large as possible; (3) the picture should be easily interpretable.

Many different display methods have been proposed in literature [9, 15–19]. Some of them will be briefly discussed here.

Pairwise scatter plots of some or all variables against each other. These plots show relations between the variables. With growing number of variables the number of possible plots grows unmanageably large. These pictures are separately simple to interpret. They contain each on their own but a fraction of the information content, but together virtually all. Distances are only partially retained.

New orthogonal axes may be chosen in the $p$-dimensional space, for example by calculating the principal components (PCs) of the data. The first principal component is an axis that is chosen so that the projections of all present points on that axis show maximum variance. The second PC is the axis that is orthogonal to the first one and contains next largest variance, and so on. The transformation of the data to scores on these new PC-axes is called the Karhunen-Loève transformation. The pairwise scatter plots of the PCs against each other are called PC-plots. As the first PCs contain, by definition, most of the variance in the data, only the first few PC-plots may suffice to depict most of the information (in the sense defined before), so a dimension reduction results. The new axes are chosen in a way to guarantee a maximum information content in the first two PCs. The only other difference with pairwise plots of variables is the interpretability, which is generally much worse, since the axes in the plots are mixtures of the original assay-axes. Insight into this mixture can be obtained from a *biplot*, a PC-plot with the original axes projected into it. This *biplot* gives information about the relation of the original axes to the PCs.

Scatter plots of variables or PCs are conceivable in three dimensions as well as in two. Grotch has reported this and similar techniques [23]. For the actual plotting, a projection of three-space on two-space is necessary, resulting in *pseudo*-three-dimensional plots. By rotating the three-dimensional space in different ways before the projection, a three-dimensional illusion may be obtained. By using advanced real-time 3D-graphics programs this illusion can be significantly enhanced, but only at high costs.

A technique that is not a simple orthogonal projection of the data onto two-space is extensively described by Kowalski and Bender [10]. This so-called non-linear mapping attempts to retain the distances between the data points. One may think of all points connected to all other points with tensionless springs and pressing this $p$-dimensional structure onto two-space. This technique is offered by ARTHUR as the NLM routine. Inter-individual distances are best retained by this method; the interpretation of other aspects than these distances is hard. A problem with this method is that an NLM-plot is dependent on the initial projection direction, so it is not uniquely determined.

Chernoff, contemplating the fact that in two dimensions a human is the best pattern recognizer, and that everyone has been studying faces right from his birth, proposed a procedure to translate every variable into a feature of a cartoon face [19 and 20]. By looking at the picture many variables are interpreted simultaneously. A problem with this technique is that not every facial feature is equally prominent, and that the ranking for recognition is subjective [21].

### Preprocessing the data

As mentioned before, the data presented quite a few gaps. This is a very usual phenomenon with retrospective medical data: a physician is not interested in all assays, so only a selection is executed. The locations of the gaps may reflect the surmises (or certainties) of the physician. If all incomplete objects are excluded from the data, it is probable that patients that are very typical for their class are lost. Patients that are easily recognized as suffering from a liver disease may not be examined for kidney failure, and so kidney function tests may not be ordered. On the other hand, omitting incomplete variables leaves only very non-specific tests. We chose deletion of all variables that missed more than 10% of the values in any class, removing the remaining gaps by deletion of objects. Nine variables and 117 objects were retained. The variables were sodium, potassium, chloride, urea, creatinine, AP, LDH, ASAT and ALAT. Excluded objects came about equally from all classes. (The arbitrariness of this way of missing data removal has stimulated us in a later study to develop another missing data handling technique, finally emerging into the method of *stepwise deletion* [24].)

Variation between different objects in the data comes from four causes. The first cause is inter-class variation (pathology): each disease has its own physiology and resulting blood concentrations. This is the difference between the patients in which we are interested. The second cause of difference between patient records is intra-class variation: this includes differences between patients suffering from the same disease. Another cause is intra-patient variation: variations of concentrations in time within a patient, the staging of the disease, as well as random or circadian fluctuations. The last source of difference is analytical error, the error made in the measurement.

The aim of class separation implies that variation from other sources than inter-class differences should be eliminated as well as possible, leaving the differences caused by pathology untouched. The usual approaches of autoscaling and class-scaling, that equalize the variation in each feature in the entire data set or per class respectively, implicitly assume that no distributional characeristics are known about the unwanted sources of variation. However, in many medical data sets, as in the one under consideration, such information is available for healthy people. For most assays, reference limits are determined on the basis of a healthy population. If the data are scaled according to the standard deviations found in this population, healthy persons will aggregate into a more or less spherical shape, depending on the distribution being symmetrical or not, and on the presence or absence of inter-assay correlations. Inter-individual and analytical variation is in this approach 'downscaled', leaving inter-class and disease-stage variation unaffected. In this *reproducibility scaling* it is assumed that analytical error and intra-individual variation are the same for both the healthy and the ill. In an earlier study by our group, reference limits were determined based on a patient population [11]. The standard deviations given in table 2 were derived from this study. The variables are divided by these numbers to get the scaled ones. Table 2 also includes the abbreviations used in the following.

Disease-stage variation is more difficult to combat. In the present study the effect of the stage of the disease is limited by choosing the moment of admittance to the hospital as reference, and sampling within 10 days. It

*Table 2. Standard deviations used for reproducibility scaling.*

| Variable | Abbreviation | s.d. |
|---|---|---|
| Sodium | NA | 2·3 |
| Potassium | K | 0·36 |
| Chloride | CL | 2·7 |
| Urea | UR | 1·4 |
| Creatinine | CR | 18 |
| *Uric acid* | *UA* | *0·067* |
| AP | AP | 26 |
| LDH | LDH | 38 |
| ASAT | ASAT | 4·7 |
| ALAT | ALAT | 6·4 |
| *Bilirubin, tot.* | *DBI* | *2·2* |
| *Bilirubin, dir.* | *TBI* | *0·60* |
| *Calcium* | *CA* | *0·11* |
| *Phosphate* | *P* | *0·22* |
| *Protein* | *TP* | *4·6* |
| *Albumen* | *ALB* | *3·9* |
| *Cholesterol* | *CHO* | *1·6* |
| *Triglycerides* | *TGL* | *0·50* |
| *Iron* | *FE* | *13* |
| *GGT* | *GGT* | *6·9* |

*Italics*: variable is deleted after missing value removal.

must be noted that for myocardial infarction this period might be too long to reduce disease-stage variation sufficiently.

For the determination of the most important variables several methods are available. The Umea group has defined [22] the Modelling Power of a variable $j$ for the model of class $c$ as being

$$MP_j = 1 - S_{j,c}^{\mathrm{mod}}/S_{j,c}^{\mathrm{tot}}$$

with $S_{j,c}^{\mathrm{mod}}$: residual standard deviation of variable $j$ in class $c$ with respect to the class model.

$S_{j,c}^{\mathrm{tot}}$ : **residual** standard deviation of variable $j$ in class $c$ without application of any model.

This *MP* emphasizes the description of a class with PC models. The discriminating powers defined by this group stress the difference between the classes. In ARTHUR a feature selection method called SELECT is available that offers a selection according to interclass/intra-class ratio of variance. SELECT, too, takes separation between the classes as its primary aim. Because we are concerned with exploration, modelling powers were calculated using SIMCA-3B. Since SIMCA-3B can elegantly cope with missing values, these calculations were done also before the reduction of the data set. In this way we get some impression of the importance of omitted variables as well. Modelling powers were calculated for models consisting of one and two PCs for each class. Some major results are summarized in table 3. It is seen that some deleted variables are very promising, although the figures must be interpreted carefully, because of the sometimes large proportion of missing data.

To investigate the value of the systematically missing variables a large study is started at our laboratory in which complete sets of routine data are prepared for every patient that is admitted to the ward for internal diseases at the Groningen University Hospital. The results are saved for later use when the patient's diagnoses are available.

### Results and discussion

First of all the *univariate statistics* of the unscaled data are calculated. The most relevant statistics are summarized in table 1. From this table it can be seen that the presence of some variables differs greatly between the diseases, as is expected because of the mentioned selectivity in ordering assays. Interesting variables are beginning to show because of differences in means between the diseases, but the optimism about their relevance is often reduced by the accompanying large standard deviations. It is noticed, that standard deviations and means are not much influenced by the deletion of missing values. This suggests that deletion of objects has been sufficiently random, since shift of means and/or standard deviation is expected if systematically some subclasses of objects were deleted.

More information may come from a bivariate approach. *Correlations* are easily obtainable. In CLAS they are calculated across the entire data set by default. The correlations found to be greater than 0.5 are reported in figure 1. They are all significant at a level of at least 99.95% (one-sided). The variables concerned turn out to form small clusters. It must be stressed that some correlations may be strongly dependent on the classes included in the data set. For instance a variable may be high for a class and normal for other classes in which another variable's level may be elevated. This situation will result in a high (negative) correlation between these variables that might be absent in a healthy control group. It follows that correlations calculated in this data set are not applicable to data-sets consisting of different classes. However, from these correlations variables that are redundant *in the context of these classes* can be detected.

It is seen that although many highly significant correlations exist, the correlations do not allow us to leave many variables out because of complete redundancy. Only the bilirubins and, to a lesser extent, creatinine and urea, and AP and direct bilirubin are roughly equivalent in this data set.

*Table 3. Largest modelling powers.*

| Class | Model size* | Data set† | Variables with largest modelling powers | | | | |
|---|---|---|---|---|---|---|---|
| Heart | 1 | F | ASAT | > | LDH | > | ALAT |
| | 1 | R | ASAT | > | LDH | > | ALAT |
| | 2 | F | ASAT | > | *GGT* | > | LDH |
| | 2 | R | ASAT | > | LDH | > | ALAT |
| Liver | 1 | F | *Bilirubin, dir.* | > | *bilirubin, tot.* | > | AP |
| | 1 | R | ASAT | > | LDH | > | ALAT |
| | 2 | F | *Bilirubin, dir.* | > | *GGT* | > | *Bilirubin, tot.* |
| | 2 | R | ASAT | > | ALAT | > | LDH |
| Kidney | 1 | F | Creatinine | > | Urea | > | *Phosphate* |
| | 1 | R | Creatinine | > | Urea | ≫ | LDH |
| | 2 | F | Creatinine | > | *GGT* | > | Urea |
| | 2 | R | ALAT | > | Creatinine | > | ASAT |

*Italics*: variable is deleted after missing value removal.
* Number of PCs in the model.
† F: full data set including data gaps.
R: reduced data set, freed from data gaps.

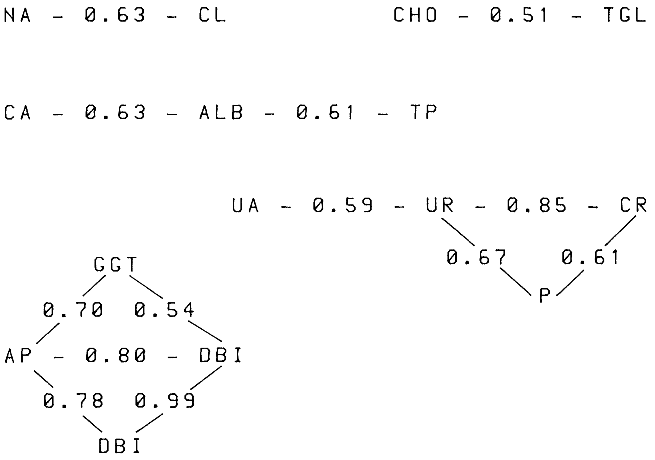

*Figure 1. Correlations in the data set that are greater than 0·5. Abbreviations from table 2.*

Plots of one variable versus another variable give visual information that may remain hidden otherwise. The modelling powers as listed in table 3 supported by clinical chemical knowledge suggest that ASAT, ALAT, LDH and creatinine may be interesting. The most informative of the set of possible plots appears to be ASAT versus creatinine. The plot is shown in figure 2. ASAT is important for the modelling of heart and liver disease groups, and creatinine is characteristic for kidney diseases. In this exploration we are concerned with description of the classes. We should be aware of the fact that for optimal separation another pair of variables might be more appropriate.

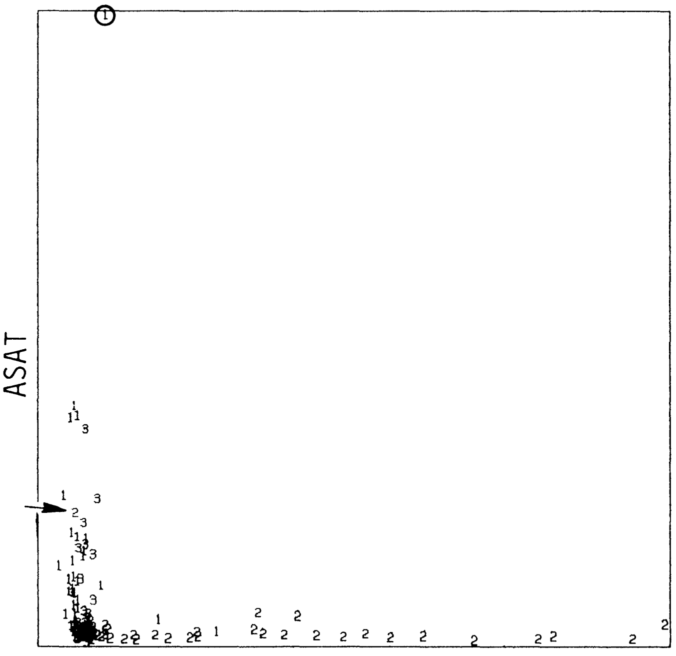

*Figure 2. ASAT plotted versus creatinine (CR) (after scaling). 1 = liver; 2 = kidney; 3 = heart.*

It is obvious from the plot that the classes are not separated completely. Patients in an early stage of illness and almost recovered patients from all disease classes join in a common region where the healthy population will be found as well. There are two apparent causes for this effect. Restricting patient samples to a period of 10 days after admittance is not enough to assure the same stage of disease, and illness is not equally manifest in all patients (for example because of therapy). Because of the rapid changes of enzyme blood concentrations after a heart attack the former phenomenon is expected to be important in the heart group. In the following we will be mainly concerned with the diverting 'tails' of the classes.

The plot shows that the KIDNEY group is well separated from the other groups, but that little separation is seen between the LIVER and HEART classes. In variable-variable plots in which best-modelling variables are chosen, this is likely to occur, unless the chosen variables are important in all models, but show different levels for each class. A KIDNEY group outlier (marked with an arrow in all figures) is clearly detected amidst HEART and LIVER patients. It should not be included in the KIDNEY class model construction. Another atypical patient, coming from the LIVER class, is noticed because of an exceptionally high ASAT level (circled in the figures). Although this patient definitely belongs to the LIVER class, the rarity of the ASAT blood concentration may disturb the construction of an adequate LIVER model. It is advisable to construct a model that does not include this patient, and therefore fits the more typical members of the LIVER group.

Another approach to bivariate plotting of the data is the use of principal components instead of variables in the plots. The plot of the first two PCs against each other is shown in figure 3. The second PC, at the abscissa, consists largely of creatinine, so KIDNEY patients spread along this axis. ASAT and ALAT are the main contributors to the first PC, therefore some similarity of this plot with the previous one is seen.

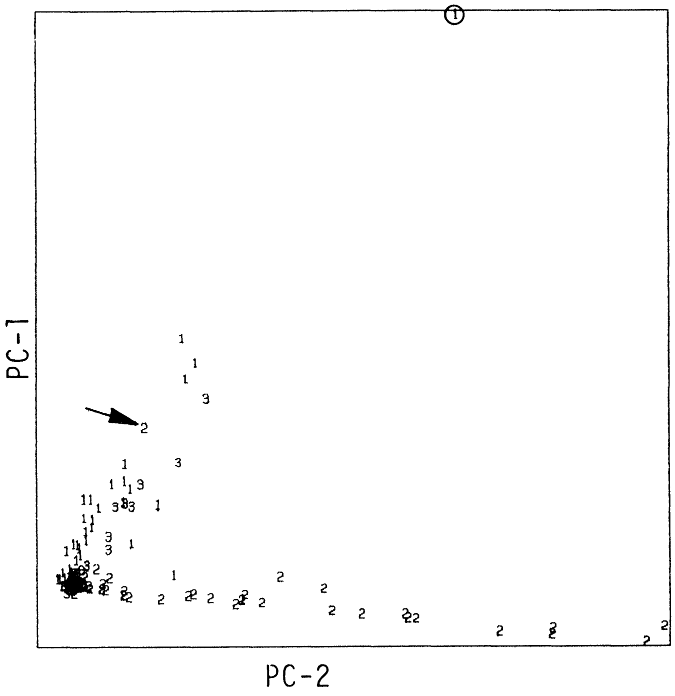

*Figure 3. PC-1 plotted versus PC-2 (after scaling). 1 = liver; 2 = kidney; 3 = heart.*

The Non-Linear Map of the data presents a different view on its distribution. The NLM plot of figure 4 is calculated with the first two eigenvectors as initial projection plane. Because of the high computational demands only a random selection of 47 patients (plus the KIDNEY outlier for illustration) is plotted. In this plot interindividual distances are best retained. KIDNEY is well separated from LIVER, but HEART is overlapped by both other classes. As only part of the data is plotted the degree of overlap might be underestimated.

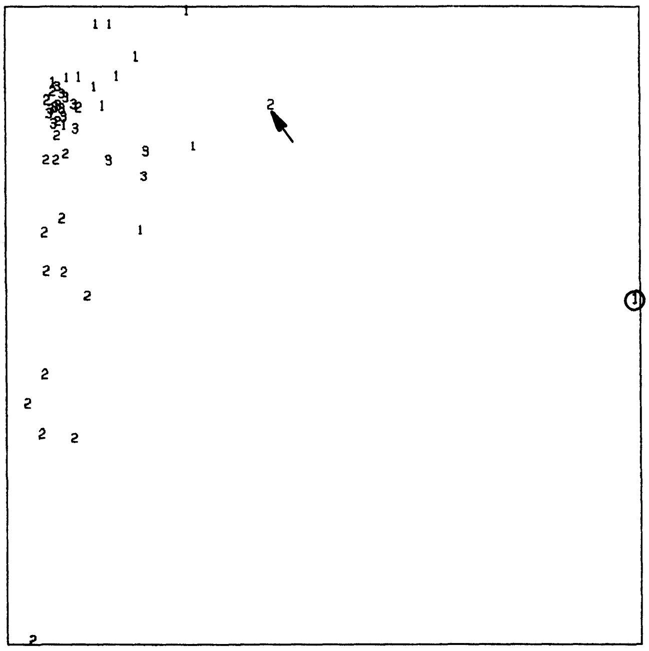

*Figure 4. Non-linear map, initiated with first two eigen vectors. 1 = liver; 2 = kidney; 3 = heart (based on only 48).*

As an extension of the bivariate plottings shown before, *pseudo*-three-dimensional plots of three variables as well as using three PCs are drawn in the figures 5 and 6. These figures are extracts of larger sets of drawings. Every complete series of plots consists of (1) an overview: the data as seen if looking along the (1,1,1) vector in the direction of the origin; (2) for each class separately a plot drawn from the same viewpoint, with the fitted PC-plane in it; (3) a rotated view on this space, one for every class, to look in a direction parallel to the PC-plane fitted to this class. Only points from the class at hand are plotted; (4) a rotated view of this space, one for every class, to look perpendicular to the PC-plane fitted to this class. Only points from the class at hand are drawn in this plot.

The selection of the variables can be based on several criteria. Using the modelling powers from table 3 creatinine-LDH-ALAT and creatinine-LDH-ASAT were most prominent. Figure 5 represents the former set. For three classes, as in the data under consideration, a complete series would result in 10 plots, of which only a selection (complete data and LIVER class) is shown. Apart from an overview, as presented by plots (*a*) and (*b*), this series provide information about the sufficiency of the two-dimensional PC-models (*c*), and the distribution within the model (*d*). If a line or point shape model would be sufficient for the data, this will be apparent in plot (*d*).

From these plots it can be seen that the classes are largely separable, but that they meet and overlap in a central part, the place where the healthy population would be found. In figure 5(*b*) the LIVER class is drawn separately. A two-dimensional PC model is tentatively fitted to it. This plot gives some insight into the relative position of the LIVER group. Plot 5(*c*) offers a view on the LIVER class parallel to the PC-plane. Deviations from the model can be judged. Apart from two patients a two dimensional model seems to be adequate in this space. In plot 5(*d*), which offers a view perpendicular to the class model, the usefulness of a two-dimensional model as opposed to a one-dimensional one can be judged. In our opinion both dimensions are sufficiently 'used' to retain them. In this way an impression can be obtained of the validity of the class model, and of its location in space relative to the other classes. As only the central part of the plot is drawn the KIDNEY outlier is not seen. The other atypical patient (from the LIVER class) is apparently less extreme in this plot (without ASAT) than was seen before. However, plot 5(*d*) shows him to be rather eccentric.

The series of plots numbered 6 gives the same views on the data as those from figure 5, but this time the three-dimensional space spanned by the first three PCs calculated from the whole data set is used to look at. The conclusions are about the same. The usefulness of (at least) two dimensions for the LIVER class model shows even more pronounced. In figure 6(*a*) the KIDNEY outlier detected earlier is caught again. The loss as compared to the previous plots is the interpretability: the axes are combinations of the original axes and cannot be named easily. The gain is that the directions that contain most variance in the data are presented in the plot. If class distinction is the major source of variance a better separation between classes may be seen in these PC-plots than in the set of variable plots.

To interpret simultaneously as much of the multivariate information as possible, faces were drawn according to a variation on the Chernoff faces by Frith [21]. As the selection of variables (a maximum of nine) to be translated to face features might be crucial for the recognition, the first nine PCs of the entire data set were used instead of the original variables. In this way at least most of the variance is present in the pictures. Most of these faces were very difficult to classify. Therefore only five stereotypical faces from each class are portrayed in figure 7. These faces were selected as stereotypical using the other techniques mentioned above: the patients come from the tails of the 'class clouds'. Note that these patients are not the most typical ones, but, rather, the most extreme cases. Even so, LIVER and HEART appear to be similar while only the 'extreme' KIDNEY patients differ reasonably from them. The distinction that is visible is similar to the distinction in the PC plot (figure 3). Thus the PCs numbered three and higher do not seem to have much influence.

Also shown in figure 7 is the KIDNEY class member that resembled markedly the LIVER family (X). This patient is the outlier that was detected also in previous displays.

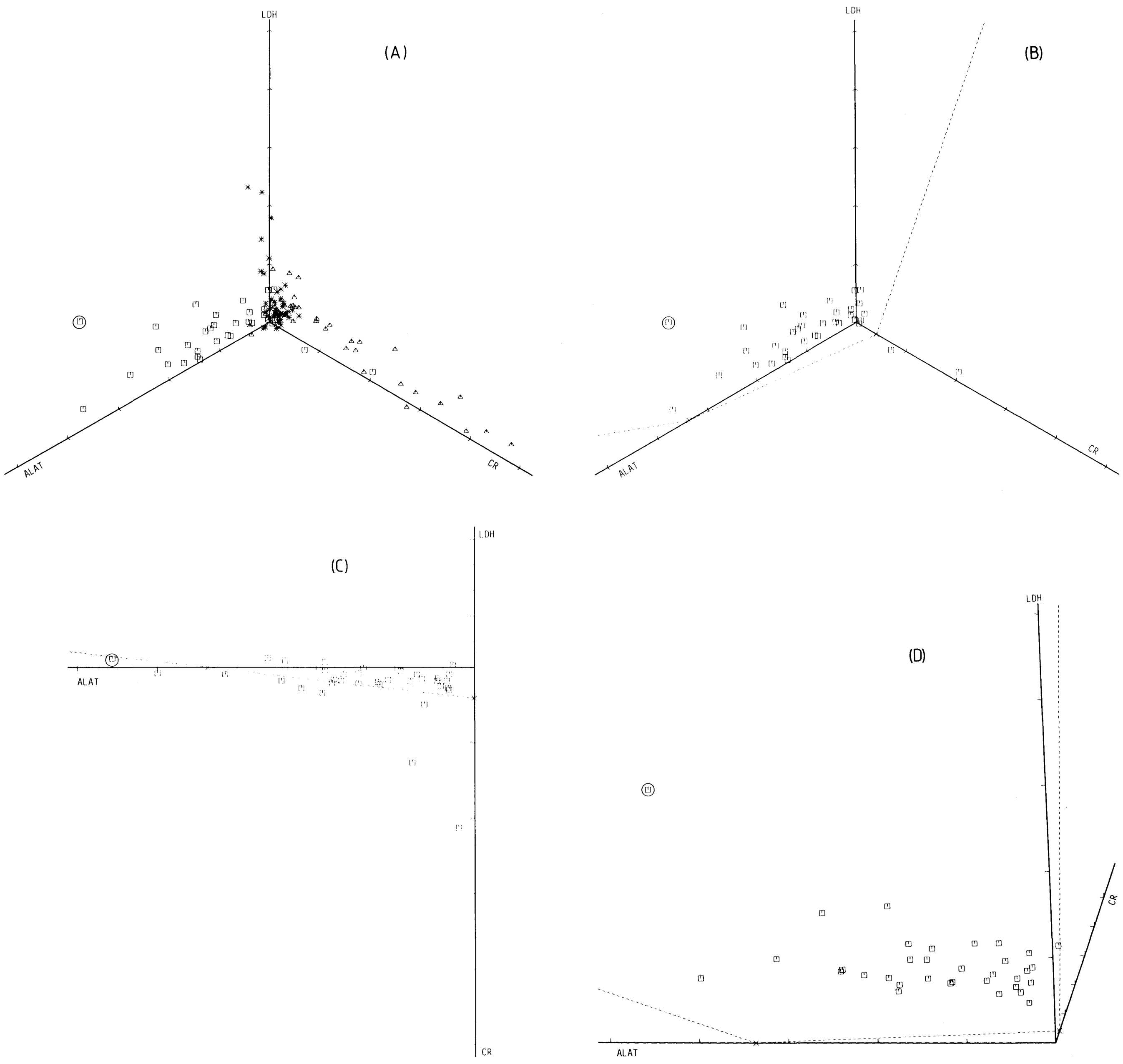

*Figure 5. Pseudo-3D plots, using ASAT, LDH and creatinine (CR) (all scaled) as the axes.*

*□ = liver; △ = kidney; * = heart.*
*(a) Overview of complete data.*
*(b) Only class LIVER, from same viewpoint.*
*(c) Only class LIVER, in parallel view on PC-plane of LIVER class.*
*(d) Only class LIVER, in perpendicular view on PC-plane of LIVER class.*
*To improve readability the axes are cut at 50 (scaled).*

The atypical LIVER patient is denoted with Y. This face is seen to differ considerably from all other faces shown here. The patients that are not 'portrayed' in figure 7 have faces that are somewhere in between the shown pictures.

It is possible that another selection of variables to be included in the face features improves the distinction, but as long as it is not known which features contribute most to visual recognition, even with an optimal set of variables, all permutations must be tried in order to identify the best clustering ordering of the set.

### Conclusions

The aim of this study was to get an impression of the separability of HEART, LIVER and KIDNEY patients on basis of 20 routine assays, and also of the presence of atypical cases in the data, and of the applicability and homogeneity of low dimensional class models. Since separability is not the only aim, the selection of variables should not be based on discrimination alone. If discrimination between classes is the leading guide for an exploration, the selection of the variables to be plotted may be biased. These plots are not characteristic for the

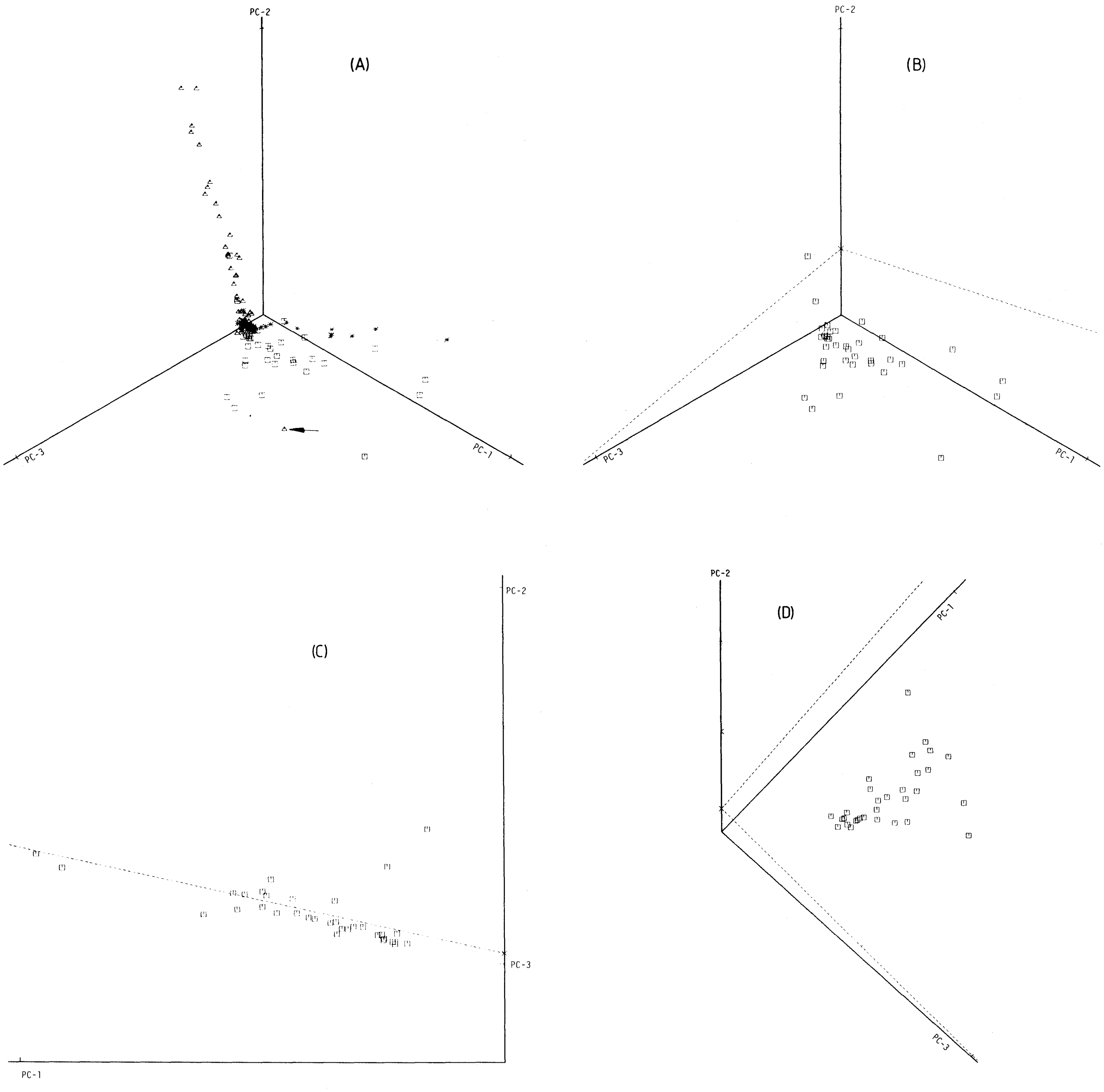

*Figure 6. Pseudo-3D plots, using PC-1, PC-2, and PC-3 (after scaling) as the axes.*
□ = *liver;* △ = *kidney;* * = *heart.*
*(a) Overview of complete data.*
*(b) Only class LIVER, from same viewpoint.*
*(c) Only class LIVER, in parallel view on PC-plane of LIVER class.*
*(d) Only class Liver, in perpendicular view on PC-plane of LIVER class.*
*To improve readability the axes are cut at 50 (scaled).*

class models and their distances. Atypical objects cannot be objectively recognized. In this paper, therefore, views on the data are chosen that stress class characteristics rather than class differences. The possibility of classification may be estimated conservatively.

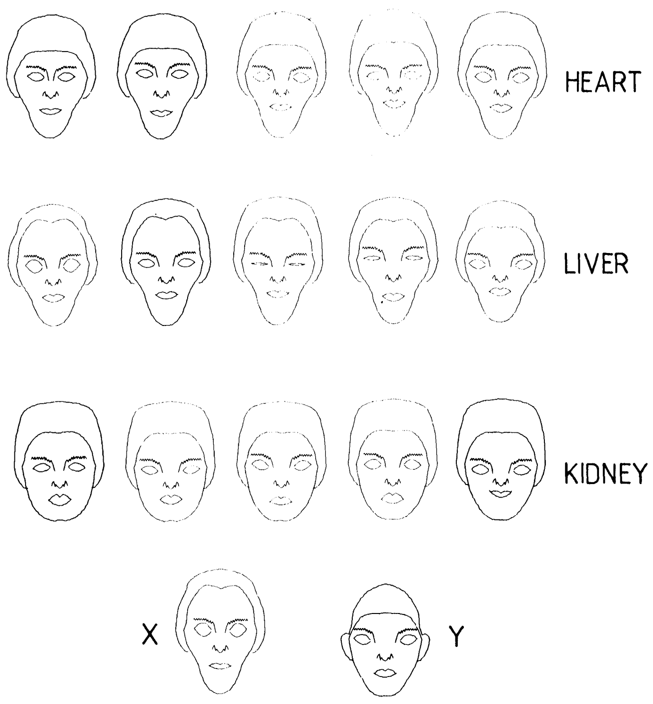

*Figure 7. Representation of patients from the HEART, LIVER and KIDNEY classes as faces.*
*X: outlier from KIDNEY class.*
*Y: atypical LIVER case.*

The selection of variables for the displays is largely based on modelling powers as calculated by SIMCA-3B. These calculations accommodate missing values. Some variables that had to be omitted because of missing values nevertheless contain valuable class modelling information. So a complete data set in which these variables are included can be expected to offer more informative displays. A large complete data set is being built now to investigate the use of the systematically missing variable scores.

Techniques that offer simultaneous display of three dimensions give more insight into the data than two-dimensional displays. It turns out to be easier for a human to combine these *pseudo*-three-dimensional plots in his mind to a single image than a set of two-dimensional plots.

If the properties of the classes in relation to the assays (variables) is the subject of the study, *pseudo*-3-D plots with three assays as basis serves interpretability. On the other hand, a more complete image of the separability is likely to result from 3-D plots of a three-PC space.

All graphical techniques make atypical values easily detectable.

As a means for data exploration, pictures of faces do not turn out to be particularly useful. Outliers may be easily recognized, but clustering of the classes is hardly visible in the analysed data set. There is little theory available for ranking of features according to 'recognizability'.

For patient classification it is necessary that all other sources of variance than inter-class variation (pathology) are removed. Reproducibility scaling based on standard deviations in a population of healthy persons reduces analytical error and random intra-patient and inter-patient fluctuations. Differences between patients that are in a different stage of their illness are not sufficiently reduced by sampling patients within 10 days of admittance. This is especially relevant with patients from disease groups with rapidly changing patterns (for example myocardial infarction). This is a serious problem which gives classification of relatively stable disease groups, and clearly staged diseases, the best chance for success. Patients under effective therapy are not always recognized as ill; it is a matter of opinion if they should be. Both effects cause the data set to be shaped like a spider, with a large proportion of seemingly 'healthy' patients, and offshoots with the more seriously ill ones.

### Acknowledgements

The authors would like to thank Professor Dr C. H. Gips, Professor Dr G. K. van der Hem, and Dr J. W. Viersma for kindly giving access to their patient data.

### References

1. Van der Voet, H., Doornbos, D. A., Meems, M. and Van de Haar, G., *Analytica Chimica Acta,* **159** (1984), 159.
2. Kryger, L., *Talanta,* **28** (1981), 871.
3. Coomans, D., Broeckaert, I., Jonckheer, M. and Massart, D. L., *Methods of Information in Medicine,* **22** (1983), 93.
4. Solberg, H. E., *CRC Critical Reviews in Clinical Laboratory Sciences* (1978), 209.
5. Coomans, D., Jonckheer, M., Massart, D. L., Broeckaert, I. and Blockx, P., *Analytica Chimica Acta,* **103** (1978), 409.
6. Van Vark, G. N., van der Sman, P. G. M., *Z. Morph. Anthrop.,* **73** (1982), 21.
7. Wold, S., Albano, C., Dunn III, W. J., Esbensen, K., Hellberg, S., Johansson, E. and Sjöström, M., in: Martens, J. (Ed.), Proceedings of the IUFOST Conference, *Food Research and Data Analysis* (Applied Science Publishers, London, 1983).
8. Varmuza, K., *Pattern Recognition in Chemistry* (Springer-Verlag, Berlin, 1980), 97.
9. Tukey, J. W., *Exploratory Data Analysis* (Addison-Wesley, Reading, Massachusetts, 1977).
10. Kowalski, B. R. and Bender, C. F., *Journal of the American Chemical Society,* 95/3 (1973), 686.
11. Hemel, J. B., Hindriks, F. A. and Van der Slik, W., *Journal of Automatic Chemistry,* **7** (1985), 20.
12. Wold, S., *Pattern Recognition,* **8** (1976), 127.
13. Harper, A. M., Duewer, D. L., Kowalski, B. R. and Fasching, J. L., in Kowalski, B. R. (Ed.), Chemometrics: Theory and Application, ACS Symposium Series 52 (American Chemical Society, Washington D.C., 1977), p. 14.

14. Hemel, J. B. and Van der Voet, H., *Analytica Chimica Acta,* **191** (1986), 33.
15. Chambers, J. M., Cleaveland, W. S., Kleiner, B., and Tukey, P. A., *Graphical Methods for Data Analysis* (Wadsworth International Group, Belmont, Duxbury Press, Boston, 1983).
16. Wang, P. C. C. (Ed), *Graphical Representation of Multivariate Data* (Academic Press, New York, 1978).
17. Everitt, B., *Graphical Techniques for Multivariate Data,* (Heinemann, London, 1978).
18. Chi-Hsiung Lin and Hwa-fu Chen, *Analytical Chemistry,* **49** (1977), 1357.
19. Chernoff, H., *Journal of the American Statistical Association,* **68** (1973), 361.
20. Bruckner, L. A., in Wang, P. C. C. (Ed), *Graphical Representation of Multivariate Data* (Academic Press, New York, 1978), p. 93.
21. Everitt, B., *Graphical Techniques for Multivariate Data* (Heinemann, London, 1978), p. 87.
22. Albano, C., Blomqvist, G., Coomans, D., Dunn III, W. J., Edlund, U., Eliasson, B., Hellberg, S., Johansson, E., Nordén, B., Joknels, D., Sjöström, M., Wold, H. and Wold, S., in Höskuldsson A. and Esbensen, K. (Eds), Proceedings of the Symposium on Applied Statistics (NEUCC, RECAU, RECKU and Danish Society of Theoretical Statistics, Copenhagen, 1981).
23. Grotch, S. L., in Kowalski, B. R. (Ed), *Chemometrics. Mathematics and Statistics in Chemistry* (D. Reidel, Dordrecht, Boston, 1984), p. 439.
24. Hemel, J. B., Van der Voet, H., Hindriks, F. R. and Van der Slik, W., *Analytica Chimica Acta,* **193** (1987), 255.

**THERMAL ANALYSIS IN RESEARCH AND PRODUCTION – AN INTENSIVE SHORT COURSE**

*To be held from 16 to 18 May in Basel, Switzerland*

Key topics are:

Instrumentation
Measurable parameters
Organic compounds
Polymers
  Thermoplastics
  High temperature polymers
  Elastomers
  Thermosets
  Fibres
  Composites
  Copolymers, blends and polymer miscibility
  Compounding selection of additives
Pharmaceuticals
Inorganics
  Catalysts
  Ceramics and glasses
  Metals and alloys
  Semiconductors
  Superconductors
Stability determination
Aging of materials
Hazards evaluation
Optimizing processing conditions
Energy
Quality control
Problem solving

The faculty include:

Patrick K. Gallagher (AT&T Bell Laboratories, USA)
Daniele Giron (Sandoz Ltd, Switzerland)
Edith A. Turi (Polytechnic University, USA)
Robert A. Weiss (University of Connecticut, USA).

On 17 May there will be a commercial exhibition combined with a social hour and consultation.

Each registrant will be provided with a workbook containing the copies of the projected slides and the recommended bibliography. Each will have the opportunity to discuss informally with the lecturers problems of their particular interest.

*For further information, contact Prof. Edith A. Turi, Polytechnic University, 5 Oxford Drive, Livingston, New Jersey 07039, USA, or the Technomic Publishing AG, Elisabethenstrasse 15, CH-4051 Basel, Switzerland.*

